# Current organization of specialist pulmonary hypertension clinics: results of an international survey

**DOI:** 10.1177/2045894019855611

**Published:** 2019-06-07

**Authors:** Carolyn Doyle-Cox, Gail Nicholson, Traci Stewart, Wendy Gin-Sing

**Affiliations:** 1 University of Ottawa Heart Institute, Ottawa, ON, Canada; 2 Alberta Health Services, Calgary, AB, Canada; 3 Heart and Vascular Center, University of Iowa, Iowa City, IA, USA; 4 Hammersmith Hospital, Imperial College Healthcare NHS Trust, London, UK

**Keywords:** health education/disease prevention/patient education, health policy, financing, and organization, pulmonary arterial hypertension

## Abstract

Optimal pulmonary hypertension (PH) management relies on a timely, accurate diagnosis and follow-up in specialized clinics by multidisciplinary teams that have clearly defined responsibilities and protocols. Internationally agreed criteria for expert center staff are lacking, particularly with respect to nurses, who often act as a central component of the team. This survey aimed to evaluate the current organization of PH clinics and the role of nurses. The survey (35 questions) was online February–December 2015 and was advertised at international PH nurse meetings and through international PH organizations to their corresponding clinics. In total, 126 healthcare professionals from 32 countries responded. According to respondents, 54% of clinics managed >200 patients, of whom 49% had a pulmonary arterial hypertension (PAH) diagnosis, on average. In terms of staff, 66% had a dedicated program administrator, 35% had one full-time nurse coordinator/practitioner/specialist, and 57% had a nurse attend outpatient clinic alongside a physician. Crucially, not all centers had a nurse in their team. The role of a nurse coordinator/practitioner/specialist varied with 51% taking patient histories/examinations and 66% managing outpatients. In 34% of clinics, nurses were involved in their own research. Protocols were available for PH therapies (81%), management of heart failure (37%) and pain (26%), and referring patients who did not have PAH/chronic thromboembolic PH back to their specialist (62%). Not all clinics are meeting all of the standards outlined in the latest guidelines with key areas of improvement being level of support from/for nurses, clear protocols, and referral pathways.

## Introduction

Pulmonary hypertension (PH) is a progressive, severely debilitating, and incurable disease characterized by increased pulmonary vascular resistance that ultimately leads to right heart failure and death.^1^ Recent estimates suggest a global prevalence of approximately 1% of adults and 10% of people aged >65 years.^2^ PH can be challenging to diagnose since it presents with non-specific symptoms and is difficult to differentiate from several other cardiopulmonary conditions.^3,4^ The World Health Organization (WHO) defines PH as a mean pulmonary artery pressure (mPAP) of at least 25 mmHg. Several subtypes have been identified:^5^ the more specific pulmonary arterial hypertension (PAH) or PH arising as a result of left-sided heart disease, lung disease or hypoxia, chronic thromboembolic pulmonary hypertension (CTEPH), or from multifactorial mechanisms.

Five-year survival rates are increasing and have reached nearly 60% in patients with PAH and 54% in other subtypes.^6^ Treatment varies according to type of PH^5^ and symptom severity^7^ and is not limited to pharmacological interventions but requires a multidisciplinary approach that includes social workers, psychologists, and palliative care teams.^8^ PH nurses play an increasingly important role and are often best placed to coordinate care; they frequently fulfill the role of PH program coordinator providing education and advice on disease state, treatments, side-effect management, and goal setting.^8^

It is logical to assume that patients will achieve better outcomes when the multidisciplinary components of specialized clinics work together efficiently and effectively as recommended in the latest guidelines.^9^ Such multidisciplinary approaches have been successful in several therapy areas, including heart failure,^10^ diabetes,^11^ chronic kidney disease,^12,13^ and cancer.^14^ The facilities and skills required for an expert referral center were published in the 2015 ERS/ESC guidelines.^9^ In the UK, the nationally designated PH centers are obliged to adhere to defined standards of care and are audited on an annual basis.^15^ Criteria for accreditation of PH centers in the USA can also be found on the Pulmonary Hypertension Association website,^16^ but there remains to be a universally agreed upon definition of an expert PH center.^4^ Nonetheless, these criteria outline the services expected of a specialist clinic and, importantly, include the establishment of clinical management protocols and a continued commitment to clinical research. However, the role of individual healthcare professionals (HCPs) within this framework is less clear, particularly for nursing/coordinating/allied health professional staff. A cohesive multidisciplinary team (MDT) with clearly defined roles, responsibilities, guidelines and recommended protocols ensures the best care for patients in accordance with the latest evidence-based recommendations. The extent to which PH clinics have achieved this is currently unclear.

This survey aimed to evaluate the current organization of PH clinics and the role of nursing/coordinator/allied health professional staff. It was hoped that the results of this survey would identify examples of best practice and any areas requiring improvement. This would enable clarification of these areas in future recommendations for coordinating and training a MDT of HCPs or for a clinic becoming accredited as an international center of excellence.

## Methods

The questionnaire (Supplemental material) was adapted from a similar survey of heart failure clinics by the authors.^17^ The survey was made available in English, French, Spanish, Russian, Farsi, Korean, Italian, German, and simplified Chinese characters. The survey was made available online between February and December 2015. The web address (www.All4PH.com) was advertised at the following international PH nurse meetings and through international PH organizations to their corresponding clinics: Pulmonary Hypertension Professional Network;^18^ PH Clinicians and Researchers;^19^ and the Bayer International PH Nurse meetings. International PH organizations were identified from the World PH Day website (http://worldphday.org). Shortly after the survey was launched, respondent feedback resulted in the following logical changes to the survey: (1) “Other” was included as a choice of answer for question 1; and (2) the lowest answer available for questions 30, 31, and 32 was changed from “5–10%” to “0–10%.” The survey was taken offline once the frequency of response was low enough to suggest that the majority of staff from clinics willing to complete the survey had done so.

The survey comprised 35 questions on patient demographics, clinic information, human resources, collaborative practice, nursing practice, and PAH management (Supplemental material).

## Results

### Respondents

A total of 126 HCPs from 32 countries across Europe, Asia, Australia, and the USA responded. No duplicate responses (i.e. HCPs from the same clinic) were identified.

### Number of patients and physicians in the clinics

The number of patients and physicians attending the clinics surveyed is summarized in Table 1. Over half (54.4%) of the clinics managed >200 patients. The majority of clinics had ≤100 new referrals per year (77.1%) and had one (33.3%), two (18.8%), or three (16.7%) PH physicians working full time in their program.

**Table 1.** Number of patients and physicians in the clinics.

Question	Responses (n)	Possible responses	Respondents (n (%))	
Average number of patients seen per week	123	<25	65 (52.8)	
		25–50	43 (35.0)	
		50–75	10 (8.1)	
		75–100	2 (1.6)	
		>100	3 (2.4)	
Current number of all patients in your program/service	90	<25	4 (4.4)	
		25–50	7 (7.8)	
		50–100	13 (14.4)	
		100–200	17 (18.9)	
		200–400	31 (34.4)	
		400–600	8 (8.9)	
		>600	10 (11.1)	
Number of new referrals per year	96	<50	36 (37.5)	
		50–100	38 (39.6)	
		100–200	9 (9.4)	
		200–300	8 (8.3)	
		300–400	5 (5.2)	
		400–500	0 (0.0)	
		>500	0 (0.0)	
Number of PH physicians in the program by clinic	96		FT	PT
		1	32 (33.3)	13 (13.5)
		2	18 (18.8)	11 (11.5)
		3	16 (16.7)	13 (13.5)
		4	5 (5.2)	9 (9.4)
		5	2 (2.1)	3 (3.1)
		>5	6 (6.3)	6 (6.3)

FT, full time; PH, pulmonary hypertension; PT, part time.

### Patient demographics

Patient demographics are summarized in Table 2. The majority (78.3%) of clinics perform long-term follow-up of patients from all WHO classifications. Overall, 96.7% of clinics had more female than male patients.

**Table 2.** Patient demographics.

Question	Responses (n)	Responses	Respondents (n (%))
Population followed long term in program/service (including pediatric)	92*	All groups	72 (78.3)
		PAH and CTEPH	32 (34.8)
		PH and pre-transplant	18 (19.6)
		PH and pre- and post-lung transplant	15 (16.3)
		Only PAH group 1	12 (13.0)
		PH and post-transplant	3 (3.3)
Percentage of patient population^†^ in each clinical classification group	90	Group 1 PAH	49.0
	83	Group 2 heart disease	21.0
	86	Group 3 lung disease	18.0
	86	Group 4 CTEPH	10.0
	75	Group 5 miscellaneous	6.0
Median patient age (years)	92	0–18	8 (8.7)
		18–60	82 (89.1)
		>60	2 (2.2)

* The total number of respondents who gave an answer for any part of the question.

† The mean percentage reported by respondents.

CTEPH, chronic thromboembolic pulmonary hypertension; PAH, pulmonary arterial hypertension; PH, pulmonary hypertension.

### Clinic resources

Of the 125 respondents who answered the question relating to the location of their clinic, 67% were located in an academic center/teaching hospital, 19% were located in a private clinic/office, and 8% were located in a community hospital. The other 6% of clinics included a regional hospital and a dedicated cardiovascular hospital.

**Fig. 1.**
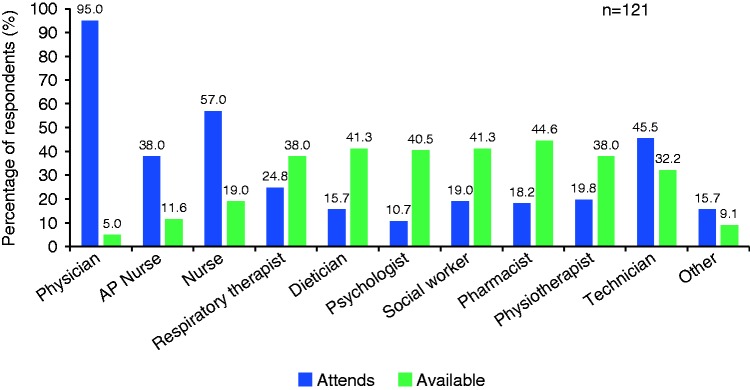
Healthcare practitioner response to the question “who attends/is available for the PH clinic?” AP, advanced practice.

A total of 80 of 121 responders (66.1%) stated their clinic had a dedicated program clerk/secretary/administrator. Figure 1 summarizes the HCPs who are available or able to attend clinic. In total, 76% of respondents reported that a nurse attends or is available at their clinic, with 49.6% respondents reporting that an advance practice nurse attends or is available. Between 51% and 63% of respondents reported that specialist HCPs—such as dieticians, psychologists, social workers, pharmacists and physiotherapists—attend or were available at their clinic. “Other” was selected as an answer by 24.8% respondents, who subsequently clarified the professions, which included medical assistants, research staff, team administrators, palliative care teams, a play therapist, a sophrologist (sophrology is a structured method involving physical and mental exercises to improve patients’ wellbeing),^20^ coordinator, and students.

**Fig. 2.**
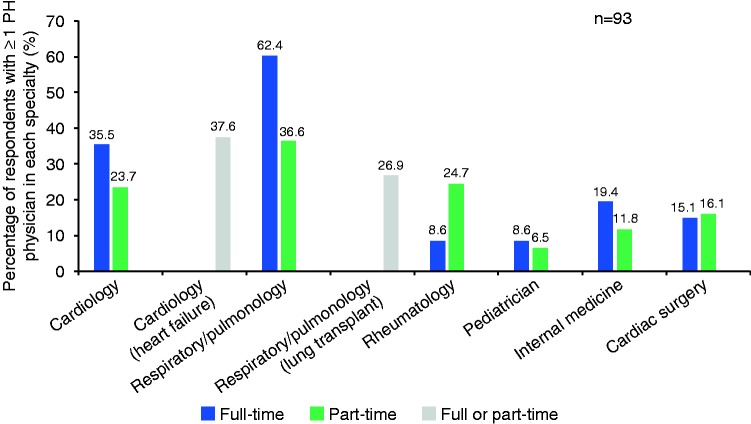
Primary specialty of a pulmonary hypertension physician.

The specialty of the PH physician(s) at the surveyed clinics is summarized in Fig. 2. The most common specialties were cardiology and respirology/pulmonary; a smaller number of clinics had PH physicians who specialized in rheumatology, pediatrics, internal medicine, or cardiac surgery.

Of 96 complete answers, 13% responded that their clinic was involved in investigator-driven clinical research, 7% in pharmaceutical clinical research, 71% in both, and 9% did not participate in clinical research. One-third of respondents (34%) reported that nurses were involved in their own research at their clinics.

### Role of the nurse coordinators/practitioners/specialists

**Fig. 3.**
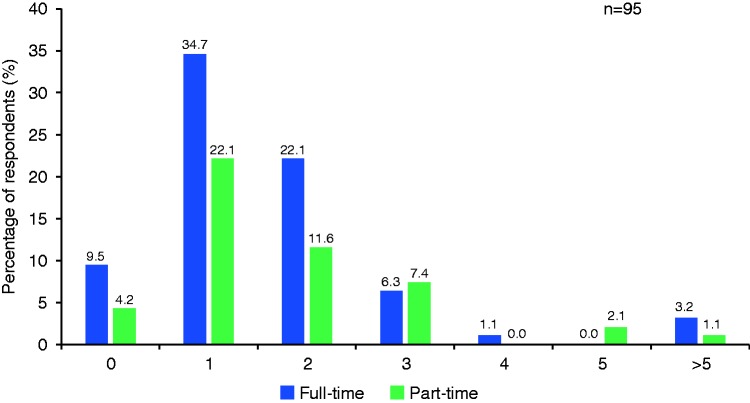
Number of nurse coordinators/practitioners/specialists employed at pulmonary hypertension clinics.

Based on responses from 95 participants, the majority of clinics had one (34.7%) or two (22.1%) nurse coordinators/practitioners/specialists working full time in their clinic, while 9.5% had none (Fig. 3). Based on responses from 95 participants, 20% and 23% responded that their clinic did not have a nurse coordinator/practitioner/specialist who attends the outpatient clinic or inpatient unit, respectively. When considering support available for hospitalized inpatients, 15% of nurse coordinators/practitioners/specialists were available to counsel only, 53% were available to counsel and assist with orders, and 44% were available to educate on management of infused therapy.

**Fig. 4.**
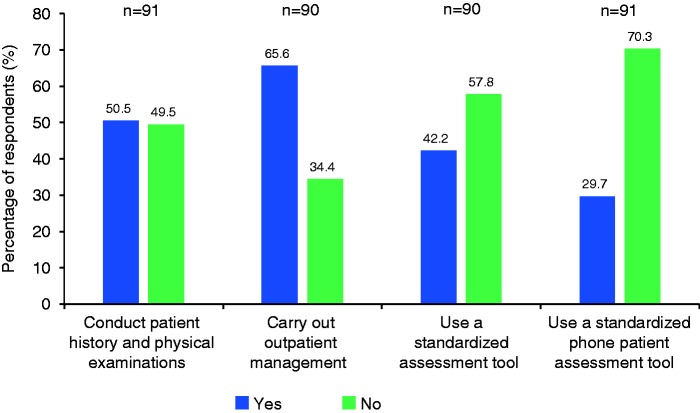
Role of nurse coordinators/practitioners/specialists.

**Fig. 5.**
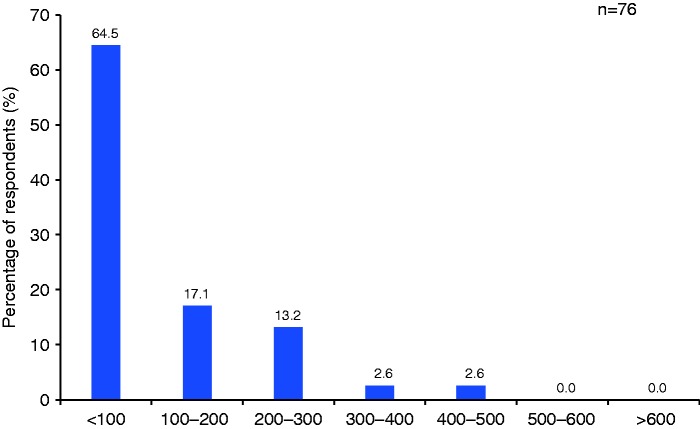
Number of phone visits per month.

The role of nurse coordinators/practitioners/specialists is summarized in Fig. 4. Approximately half of respondents stated the role of the nurse coordinators/practitioners/specialists included taking patient history and physical examinations and 66% of respondents stated that they managed outpatients. A standardized patient assessment tool was used by nurses in 42% of clinics and a standardized phone patient assessment tool was used by 30% of clinics. The number of phone contacts per month is summarized in Fig. 5; according to respondents, the majority of clinics made <100 phone contacts per month with only four clinics making >300, and none making >500 phone contacts per month.

### Patient education

Of 87 complete answers, 89% responded that their clinic did initiate and were responsible (directly or indirectly) for ensuring prostacyclin therapy patient education with 62% sharing this service with a specialty pharmacy, 53% performing this service via follow-up phone calls between visits, and 24% of clinics only providing this service in clinic/hospital. According to respondents (n = 88), 77% of clinics offered heart failure education, with 42% doing so via follow-up phone calls between visits and 35% offering it only in outpatient clinics. Self-care or living with PH support was offered by 88% of clinics, with 52% providing it via follow-up phone calls between visits and 36% offering it only in outpatient clinic. Of 88 complete answers, 91% responded that their clinics coordinate referral to other consultant services.

### Therapeutic treatment and clinical management of PH in clinics

The majority (65.1%) of respondents answered that 11–40% of patients were receiving monotherapy, while 73% of respondents stated that 21–60% of patients were on dual therapy, and 49% of respondents stated that ≤10% of their patients were on triple therapy. Of 89 complete answers, respondents reported that protocols were available for PH therapies (81%), heart failure management (37%), and pain management (26%), with 12% of clinics reportedly without any protocols for medical therapies. Of 86 complete answers, 62% of clinics did not have a protocol for referring patients who were not diagnosed with PAH or CTEPH back to their specialist or GP. Based on responses of 96 HCPs, 31% of clinics did not work with a palliative care group/physician, 42% provided this service for both inpatients and outpatients, while 24% and 3% only provided this for inpatients and outpatients, respectively.

## Discussion

To our knowledge, this international survey is the first to gather information—unrestricted by PH group or HCP role—on the organization of PH clinics, with a focus on the role of nurses. Previous international surveys have investigated physician-reported (n = 496) management of PAH and CTEPH in specialist PH centers.^21,22^ However, these studies only included clinics that met the 2009 ESC/ERS criteria for a PH center (i.e. managed ≥50 patients with PAH or CTEPH; received ≥2 new referrals each month; performed ≥20 vasoreactivity tests each year; participated in clinical research; had a MDT and direct access to other medical programs). As such, they were not able to comment on the extent to which PH clinics generally are meeting these criteria. Therefore, the present findings are the first global assessment of the administration of PH clinics (n = 126), according to their staff.

A major recommendation of the 2015 ESC/ERS guidelines is that patients with PAH and CTEPH are treated at an expert referral center. Specifically, as a minimum, the 2015 ESC/ERS guidelines state an expert referral center should manage ≥50 patients with PAH or CTEPH and receive ≥2 new referrals each month of patients with documented PAH or CTEPH. The number of patients managed by centers is recommended to be ≥200, of which half should have a final diagnosis of PAH.^9^ In this survey, 54% of respondents stated that the total number of patients enrolled at their clinics exceeded 200 and that the average percentage of patients with PAH was 49%. As the awareness of PH has increased in recent decades,^23^ it is perhaps not surprising that 8.9% of clinics manage 400–600 patients. As the population of PAH patients grows, the demand for specialized clinical support will increase.

The criteria for an expert referral center also includes the presence of a well-coordinated MDT;^9^ PH nurses are key to achieving this.^8^ It is therefore surprising, given the indisputable value of this role, that 10% of clinics did not have a full-time PH nursing/coordinator/allied health professional at all and 35% only had one. Other concerning results included the fact that only 57% of programs had a nurse attend outpatient clinic alongside a physician and only 66% had a dedicated clerk/secretary/administrator for the program. This suggests that continuity of care might suffer and is particularly worrying considering the high volume of patients reported by staff from some of the clinics.

Palliative care support is essential for patients with PH, as there is no known cure, but the concept of palliative care is often misunderstood.^24,25^ It is often thought to refer specifically to end-of-life care by a specialist team within hospice care,^24,25^ but palliation of symptoms and goals of care conversations should actually be introduced early in the disease trajectory by clinicians as part of routine management of PH.^26,27^ The latest guidelines for management of PH also recommend involvement of a specialist palliative care team where appropriate,^9^ but an approach to palliative care—and timing to referral—has not been agreed upon. Results from a small retrospective UK study, in which the suitability for palliative care was determined according to patients’ prognostic indicators and clinical course before death, showed that only 11 of 31 (35.5%) suitable patients received support from specialist care services.^25^ Results from this survey appear to be in agreement with previous findings, as almost one-third (31%) of respondents reported that they do not work with a palliative care group. However, it is unclear if this is because patients were receiving adequate support from the clinic or if this is indicative of a gap in care and suggests that further investigation is required.

When considering referral to any other team, 91% of respondents reported that their clinics coordinate referral to other specialties such as transplant and genetics services. However, as the only possible responses were “yes” or “no,” it is not clear what proportion of respondents would consider their clinic to conduct timely and appropriate referral; this also warrants further investigation. Appropriate referral to other specialists or centers relies on clear patient pathways and protocols, which guide physicians in timing and suitability of referral.

Almost all clinics had more female than male patients. The approximate ratio was not captured by this question but previous studies suggest that PH clinics that routinely manage PAH patients are likely to have a ratio of 2:1 female to male patients.^28^ Caring for women of childbearing age or who are pregnant presents a different set of challenges and therefore requires separate referral guidelines; this should also be taken into account in future PAH guidelines.

Continuing education of staff and the use of protocols is essential for an efficient and effective MDT team. It is therefore somewhat encouraging that 88% of respondents stated there were protocols available for PH therapies. However, only 37% and 26% of respondents stated there were protocols for heart failure management and pain management, respectively. As heart failure is often the ultimate cause of death, it is concerning that the majority of clinics do not have protocols in place for this situation.

One of the goals of any PH service should be to have a well-coordinated MDT with clear protocols and referral pathways, in order to improve patient care and quality of life. A lack of clear guidance on the roles and duties of each member of the team might be partly responsible for the current range in responsibilities reported for each role. This is a challenging problem to remedy, particularly with respect to nurses, since the role of a nurse differs across countries according to legislation. For example, in the UK, USA, and Canada, nurse practitioners can prescribe medications, run independent clinics, undertake physical assessments, and make referrals, but this is not the case in some European countries.^29^ While a detailed universal outline of each staff member’s role may not be a suitable solution, an ideal framework, with which to apply recommended protocols, would be helpful and one such example has been proposed.^8^ In this example, the ideal PH MDT is largely split into the PH nurse coordinator(s)/practitioner(s)/specialist(s), providers and support staff.^8^ Within this framework, the providers (physician, nurse practitioner) are primarily responsible for clinical management (e.g. diagnosis, prescribing treatment, specialist referrals), the support staff are primarily responsible for administrative tasks (e.g. phone triage, scheduling visits and procedures, processing medication refills), and the PH nurse coordinator/practitioner/specialist is responsible for coordination of care, patient advocacy, and patient liaison.^8^ Findings from this survey and future studies can be used to build on this framework. This is particularly important for advanced practice nurses, since their role differs considerably between and within countries and therapy area.^30^ Indeed, the International Council of Nurses define an advance practice nurse as “a registered nurse who has acquired the expert knowledge base, complex decision-making skills and clinical competencies for advanced practice, the characteristics of which are shaped by the context and/or country in which s/he is credentialed” and recommend a Master’s degree for entry level.^31^ Therefore, clear governance and educational strategies are an important goal for the field. The results of this study could help to develop a framework of core competencies for advanced practice nurses caring for patients with PH and once these have been established, the gaps in knowledge will be evident. Identifying these knowledge gaps could then facilitate the provision of additional training in areas such as advanced communication skills, counselling, goal setting, and motivational interviewing, through courses, preceptorship programs, and online resources for nurses.

### Limitations

This study is the first to gather information on the current organization of PH clinics, with the intention of starting a discussion on what the core competencies and responsibilities of PH nurses are; as such, this initial survey had several limitations. This survey was advertised by PH organizations to their corresponding clinics and HCPs of any role and at any PH clinic were invited to complete it. This approach has the advantage of yielding a lot of information about current management of PH clinics. However, there are several important limitations to this study: little information was collected on the respondents themselves; therefore, the professional role of respondents and any potential conflicts of interest, and how this might have affected their answers, is unknown. As this survey only collected responses from HCPs who had seen the survey advertised at PH organization events or had been contacted via PH organizations and volunteered their time, it is likely that these respondents represent the more engaged HCPs, who are most familiar with the latest recommendations whereas data from smaller, non-specialist providers of care are unlikely to have been captured. A major limitation of the survey is that it was not possible to determine the total number of HCPs who were aware of the survey but chose not to participate: the response rate cannot be determined. As with all surveys, the responses are not objective data and may be subject to recall and response bias, honesty of response, or introspective ability.^32,33^ The impact of response and self-reporting bias on this survey is more likely to be that provision of care has been overestimated in this survey, rather than underestimated. Therefore, further investigation would be required to validate the self-reported findings described herein. In addition, the number of questions with a yes/no (or similar positive/negative) answer may have limited the amount of information captured. For example, it would be more informative to have an estimate of the ratio of female to male patients in PH clinics, rather than simply there either were or were not more women than men or not, although it must be taken into consideration that changing the nature of this question would potentially introduce recall and other errors. For other questions, answers to multiple-choice questions can be difficult to interpret. For example, 78% of respondents selected the answer “All WHO groups” [are followed up long term in the program], but 35% selected the answer “PAH and CTEPH groups.” These responses appear contradictory and it is unclear whether respondents who selected both “All WHO groups” and “PAH and CTEPH” groups were simply ticking all that apply or were indicating that these two groups in particular are followed up in the program, but no single group are excluded from follow-up entirely. The contradiction may also be due to respondents interpreting the question as WHO functional class groups rather than all clinical classifications. A related limitation is the lack of a thorough pilot study. A small pilot study was run to ensure questions were understood, but in order to not limit the number of respondents, the questionnaire was distributed to only a small number of PH professionals at an international conference. As such, only minor amendments to the survey were made before survey launch; further refining of the survey may have been possible before launch had the pilot study been of a greater sample size. In this study, a small number of minor changes were made once the survey was live: these are detailed in the methodology section and have had no bearing on the results or conclusions of the study. This survey was designed to be simple and quick to complete for HCPs with little spare time in order to attract a greater number of respondents. Future audits and studies should be conducted to confirm whether these results are representative of global PH management, in general. Further studies should ensure that the patient views on the organization of their care are also taken into account.

It is worth noting that current recommendations for optimal PH program staffing and structure, including use of clinical nurse specialists (CNSs), are based on a consensus of opinion, with evidence for a positive effect on patient outcomes not yet being available in this setting.^9^ However, there is published evidence supporting the value of the CNSs in other disease areas, with nurse managed care deemed to be as effective as care managed by doctors, and patients reporting higher levels of satisfaction.^34–41^ For example, studies have shown that patients with arthritis seen by specialist nurses had a greater feeling of being able to control their arthritis and better outcomes than those seen by routine clinic staff.^34,35^ Significant improvements in patient follow-up and treatment rates were also seen following the introduction of a CNS into the team caring for patients with chronic hepatitis C.^36^ Breast cancer,^37,40^ stroke,^38^ and some adult heart failure^39^ patients receiving care from a nurse specialist also reported significantly higher levels of satisfaction than those receiving care from general practitioners.

The role of the CNS is being increasingly formally recognized within national evidence-based guidelines. For example, UK guidelines for the management of early and locally advanced breast cancer require that all patients are assigned to a named breast care nurse specialist to support them throughout diagnosis, treatment, and follow-up.^42^

## Conclusions

The results of this international HCP survey of PH clinic administration demonstrate that not all clinics are meeting all of the criteria for an expert referral center, outlined in the 2015 ESC/ERS guidelines. Key findings are the lack of nurse support and the lack of clear protocols and referral pathways at some clinics. The authors feel development of clearer recommended protocols are warranted, such as how many patients one nurse specialist should be responsible for, and what protocols and further training should be made available to staff. Ultimately, the authors hope information gathered in this initial survey can inform future discussions on the core competencies and responsibilities of PH clinic staff, particularly nurses. Through collaboration and further study, it is possible to improve care, enable more PH clinics to meet the accreditation criteria for specialist PAH centers supported by PHA^16^ and NHS England^26^ and provide further input on the guidelines and accreditation criteria for specialist PH clinics.

## Supplemental Material

Supplemental material for Current organization of specialist pulmonary hypertension clinics: results of an international survey Click here for additional data file. Supplemental Material for Current organization of specialist pulmonary hypertension clinics: results of an international survey by Carolyn Doyle-Cox, Gail Nicholson, Traci Stewart and Wendy Gin-Sing in Pulmonary Circulation
