# Supplementary material for: Current organization of specialist pulmonary hypertension clinics: results of an international survey
Source: Pulm Circ. 2019 Jun 7;9(2):2045894019855611. doi: 10.1177/2045894019855611 (PMC6557030; doi:10.1177/2045894019855611)
Supplement: Supplemental material for Current organization of specialist pulmonary hypertension clinics: results of an international survey [file Supplemental_Material.pdf]

PH Clinic Questionnaire

Instructions:

Fill in the circles that apply. Please forward survey to any PH program/service you may be aware of in your area..

Please provide email address, phone number and center location.

Your contact information will be kept separately from your survey responses

Clinic

1. Location
    - o Outpatient clinic or private office
    - o Community Hospital
    - o Academic Center/Teaching Hospital
    - o Other______
2. Average number of patients seen per week
    - o <25
    - o 25-50
    - o 50-75
    - o 75-100
    - o >100
3. Is there a dedicated clerk/secretary (administrator) for program/service
    - o Yes

- No

4. Who attends/ is available for the PH clinic
    - Physician
        - Attends
        - Available
    - Advanced Practice Nurse
        - Attends
        - Available
    - Nurse
        - Attends
        - Available
    - Respiratory therapist
        - Attends
        - Available
    - Dietician
        - Attends
        - Available
    - Psychologist
        - Attends
        - Available
    - Social Worker
        - Attends
        - Available
    - Pharmacist
        - Attends

        - Available
    - Physiotherapist (i.e. performs exercise testing)
        - Attends
        - Available
    - Technician (i.e. 6mwt, PFT’s, Echo)
        - Attends
        - Available
    - Other
        - describe
        - Attends
        - Available

5. Current number of all patients in your program/service
    - <25
    - 25-50
    - 50-100
    - 100-200
    - <200
    - 200-400
    - 400-600
    - >600
6. Percentage of patients in each clinical classification group
    - WHO group 1 – Pulmonary Arterial Hypertension ______%
    - WHO group 2 - Pulmonary Hypertension due to left heart disease_____%
    - WHO group 3- Pulmonary Hypertension due to lung disease______%

- WHO group 4 – Chronic Thromboembolic Pulmonary Hypertension______%
- WHO group 5 – miscellaneous ______%

7. Number of all new referrals per year
    - <50
    - 50-100
    - 100-200
    - 200-300
    - 300-400
    - 400-500
    - >500

8. Population followed long tem in program/service (including pediatric)
    - PH patients (All WHO groups)
    - Only PAH patients (WHO group 1)
    - PAH and CTEPH (WHO group 1 and IV)
    - PH and Lung transplant patients
        - Pre
        - Post
        - Both
9. Median age of the patient
    - 0-18
    - 18-30
    - 30-40

- 40-50
- 50-60
- 60-70
- >70

10. Female to Male Ratio
    - More females
    - More males

PH Physicians-

11. How many PH Physicians are in the PH program/service
    - 1
        - FT
        - PT
    - 2
        - FT
        - PT
    - 3
        - FT
        - PT
    - 4
        - FT
        - PT
    - 5

    - FT
    - PT
- > 5
    - FT
    - PT

12. PH Physician Specialty
- Cardiology
    - Specialty in heart failure_____
    - Number FT_____
    - Number PT_____
- Respirology/Pulmonology
    - Specialty in lung transplant_____
    - Number FT_____
    - Number PT_____
- Rheumatology
    - Number FT_____
    - Number PT_____
- Pediatrician
    - Number FT_____
    - Number PT_____
- Internal medicine
    - Number FT_____
    - Number PT_____
- Cardiac surgery

    - Number FT_____
    - Number PT_____

13. Does your PH program/service participate in clinical research
    - Yes
        - Investigator driven
        - Pharmaceutical
        - both
    - no

14. Do you work with a palliative care group/physician
    - Yes
        - In-patient only
        - Out-patient only
        - both
    - no

Nursing/Coordinator/Allied Health Professional

15. How many Nursing/Coordinator/Allied Health Professional's are in the PH program/service
    - 0
    - 1
        - FT
        - PT
    - 2
        - FT
        - PT
    - 3

    - FT
    - PT
- 4
    - FT
    - PT
- 5
    - FT
    - PT
- > 5
    - FT
    - PT

16. Is there a Nursing/Coordinator/Allied Health Professional's that attends the outpatient clinic
    - Yes
    - No

17. Is there a Nursing/Coordinator/Allied Health Professional's to see hospitalized inpatients
    - Yes
        - Counsel only
        - Counsel and assist with orders as per hospital protocols
        - Educate patients on management of infused therapy
    - no
18. Is there a research coordinator
    - Yes
        - Separate position

        - PH nurse role
- No

19. Use of Protocols /Medical Directives
    - Yes
    - No
20. Does nursing/allied health do history and physical exam
    - Yes
    - No
21. Is there a nursing assessment tool (standardized patient assessment tool)
    - Yes
    - No
22. Is outpatient management carried out by the nurse/allied health professional
    - Yes
    - No
23. Is there a phone nursing assessment tool (standardized patient assessment tool)
    - yes
    - no
24. Number of phone visits per month
    - <100
    - 100-200
    - 200-300
    - 300-400
    - 400-500
    - 500-600
    - >600

25. Is the nurse involved in his/her own research
    - Yes
    - No
26. Does your program/service initiate and train Prostacyclin therapy patient education
    - Yes
        - Shared education with Specialty pharmacy nursing
        - Follow-up phone calls between visits
        - Only in clinic-hospital
    - No
        - Send patient to larger center for education
27. Heart failure education
    - Yes
        - Follow-up phone calls between visits
        - Only in clinic
    - No
28. Self-care/living with PH support
    - Yes
        - Through phone calls between visits
        - Only in clinic
    - No

29. Coordinate/referral to other health care services/organizations
    - Yes
    - no

Pulmonary Hypertension Management

30. Estimate percentage of patients on mono therapy

    - 0-10%
    - 11-20%
    - 21-30%
    - 31-40%
    - 41-50%
    - 51-60%
    - 61-70%
    - 71-80%
    - 81-90%
    - 91-100%

31. Estimate percentage of patients on dual therapy

    - 0-10%
    - 11-20%
    - 21-30%
    - 31-40%
    - 41-50%
    - 51-60%
    - 61-70%
    - 71-80%
    - 81-90%
    - 91-100%

32. Estimate Percentage of patients on triple therapy

- 0-10%
- 11-20%
- 21-30%
- 31-40%
- 41-50%
- 51-60%
- 61-70%
- 71-80%
- 81-90%
- 91-100%

33. Estimate percentage of patients on prostacyclin therapy

- none
- 1-10%
- 11-20%
- 21-30%
- 31-40%
- 41-50%
- 51-60%
- 61-70%
- 71-80%
- 81-90%
- 91-100%

34. Are there protocols for discharging non PAH/CTEPH patients from clinic

- Yes
- No

35. Are there protocols for medical therapies

- Yes
    - PH therapies
    - HF management
    - Pain management
- No

References

1.Galie N, Torbicki A, Barst R, et al. Guidelines on diagnosis and treatment of pulmonary arterial hypertension. 2004 The Task Force on Diagnosis and Treatment of Pulmonary Arterial Hypertension of the European Society of Cardiology. *European Heart Journal*. 25(24):2243–78.

2.Badesch DB, Abman SH, Simonneau G, et al. 2007 Medical therapy for pulmonary arterial hypertension: updated ACCP evidence-based clinical practice guidelines. *Chest*. 2007;131(6):1917–28.

3.Simonneau G, Robbins IM, Beghetti M, et al. 2009. Updated clinical classification of pulmonary hypertension. *Journal of the American College of Cardiology*. 2009;54(1 Suppl):S43–54.

4.D'Alonzo GE, Barst RJ, Ayres SM, et al. Survival in patients with primary pulmonary hypertension. Results from a national prospective registry. *Annals of Internal Medicine*. 1991;115(5):343–9.

5. Komenda P, Levin A. 2006. Analysis of cardiovascular disease and kidney outcomes in multidisciplinary chronic kidney disease clinics: complex disease requires complex care models. *Current Opinionin in Nephrology and Hypertension*. 15:61-6.

6. Wright FC, De Vito C, Langer B, Hunter A. 2007. Multidisciplinary cancer conferences: a systematic review and development of practice standards. *European Journal of Cancer.* 43:1002-10.

7. McAlister FA, Stewart S, Ferrua S, McMurray JJ. 2004 Multidisciplinary strategies for the management of heart failure patients at high risk for admission: a systematic review of randomized trials. Journal of the American College of Cardiology. 44:810-9.

8. McLaughlin VV, Archer SL, Badesch DB, Barst J,FarberHW,LindnerJR,MathierMA,McGoon MD, Park MH, Rosenson RS, Rubin LJ, Tapson VF, Varga. 2009. Journal of the American College of Cardiology, ACCF/AHA Expert Consensus Document on Pulmonary Hypertension A Report of the American College of Cardiology Foundation Task Force on Expert Consensus Documents and the American Heart Association Developed in Collaboration with the American College of Chest Physicians; American Thoracic Society, Inc; and the Pulmonary Hypertension Association. J of the American College of Cardiology. 53:1573–1619.

9. François Haddad, MD, Tyler Peterson, MD, Eric Fuh, MD, et.al. 2011. Characteristics and Outcome After Hospitalization for Acute Right Heart Failure in Patients With Pulmonary Arterial Hypertension. *Circulation: Heart Failure*. 4: 692-699

Carolyn Pugliese RN, MSN
APN, Ottawa Pulmonary Hypertension Clinic

University of Ottawa Heart Institute
40 Ruskin Street
Room H 4422A
Ottawa, ON K1Y 4W7
Phone- 613-761-5396
Fax- 613-761-4327
Pager 613-274-1482
Email cpugliese@ottawaheart.ca
